# A molecular and antigenic survey of H5N1 highly pathogenic avian influenza virus isolates from smallholder duck farms in Central Java, Indonesia during 2007-2008

**DOI:** 10.1186/1743-422X-8-425

**Published:** 2011-09-07

**Authors:** Hendra Wibawa, Joerg Henning, Frank Wong, Paul Selleck, Akhmad Junaidi, John Bingham, Peter Daniels, Joanne Meers

**Affiliations:** 1 CSIRO-Australian Animal Health Laboratory, Geelong, Victoria, Australia; 2 School of Veterinary Science, The University of Queensland, Gatton Campus, Gatton, Queensland, Australia; 3 Disease Investigation Centre Regional IV Wates, Yogyakarta, Indonesia

## Abstract

**Background:**

Indonesia is one of the countries most severely affected by H5N1 highly pathogenic avian influenza (HPAI) virus in terms of poultry and human health. However, there is little information on the diversity of H5N1 viruses circulating in backyard farms, where chickens and ducks often intermingle. In this study, H5N1 virus infection occurring in 96 smallholder duck farms in central Java, Indonesia from 2007-2008 was investigated and the molecular and antigenic characteristics of H5N1 viruses isolated from these farms were analysed.

**Results:**

All 84 characterised viruses belonged to H5N1 clade 2.1 with three virus sublineages being identified: clade 2.1.1 (1), clade 2.1.3 (80), and IDN/6/05-like viruses (3) that did not belong to any of the present clades. All three clades were found in ducks, while only clade 2.1.3 was isolated from chickens. There were no significant amino acid mutations of the hemagglutinin (HA) and neuraminidase (NA) sites of the viruses, including the receptor binding, glycosylation, antigenic and catalytic sites and NA inhibitor targets. All the viruses had polybasic amino acids at the HA cleavage site. No evidence of major antigenic variants was detected. Based on the HA gene, identical virus variants could be found on different farms across the study sites and multiple genetic variants could be isolated from HPAI outbreaks simultaneously or at different time points from single farms. HPAI virus was isolated from both ducks and chickens; however, the proportion of surviving duck cases was considerably higher than in chickens.

**Conclusions:**

The 2.1.3 clade was the most common lineage found in this study. All the viruses had sequence characteristic of HPAI, but negligible variations in other recognized amino acids at the HA and NA proteins which determine virus phenotypes. Multiple genetic variants appeared to be circulating simultaneously within poultry communities. The high proportion of live duck cases compared to chickens over the study period suggests that ducks are more likely to survive infection and they may better suit the role of long-term maintenance host for H5N1. As some viruses were isolated from dead birds, there was no clear correlation between genetic variations and pathogenicity of these viruses.

## Background

Avian influenza (AI) viruses have been isolated from a wide range of avian species representing several orders [1,2]. However, AI virus isolations have been reported mostly from the orders of *Anseriformes *[3], especially from dabbling ducks (subfamily *Anatinae*), which have been detected carrying a number of H3, H4 and H6 subtype viruses, but less commonly H5, H7 and H9 viruses [4,5]. Although 16 antigenic subtypes of HA (H1-H16) and 9 antigenic subtypes of NA (N1-N9) of AI viruses have been identified [5,6], viruses from H5 and H7 subtypes have become a particular concern because they can cause severe and fatal infection in both avian and mammalian hosts, including humans [7,8]. Experimental studies showed that ducks were susceptible to the infection of some Asian H5N1 subtype viruses with varied degree of disease severity, ranging from minimal clinical signs to death, but they could continue to shed virus when surviving infection [9,10].

Since the initial H5N1 HPAI outbreaks in China in 1997, the virus has circulated continuously amongst poultry causing subsequent epidemics in several countries across Asia, Europe, and Africa [11]. In Indonesia, HPAI virus infection was announced firstly in January 2004, despite this virus was suspected to have caused deaths in chickens already since October 2003 [12]. A phylogenetic study estimated that the time of the first introduction of H5N1 virus into Indonesia was between April and July 2003 [13]. Although details of the original introduction of H5N1 into Indonesia poultry are still unclear, there is a direct precursor-descendant link between H5N1 viruses isolated from Hunan province, China in 2002 and the Indonesian 2.1 clade viruses [14]. Up until March 2011, Indonesia continued to report the majority of outbreaks in poultry worldwide, with 31 of 33 provinces in this country affected and more than 11 million chickens have died or been culled [15,16]. In some circumstances, H5N1 virus can be transmitted to humans resulting in fatal disease. Indonesia also reported the highest prevalence in humans up to June 2011 with a case fatality of 82% (146 of 178) [17].

To date, all Indonesian H5N1 viruses have been classified into clade 2.1, with three virus sublineages being present within this clade: 2.1.1, 2.1.2 and 2.1.3 [18]. The viruses within clade 2.1.1 were mainly isolated from HPAI-infected poultry during the outbreaks between 2003 and 2005. Clade 2.1.2 consisted of avian- and human-derived viruses, isolated predominantly from Sumatra between 2004 and 2007. Clade 2.1.3 comprised a range of viruses that were isolated either from birds or from humans since 2004. While clade 2.1.3 viruses have predominated and they continue to circulate in Indonesia, the number of isolated H5N1 viruses from clade 2.1.1 and 2.1.2 has substantially declined since 2005 [19]. Although 2.1.3 viruses have spread and become endemic in many provinces in Indonesia, a new sublineage virus has emerged since 2004 [19,20].

Genetic and antigenic data are important to provide more insight into the epidemiology of HPAI in Indonesia. A recent epidemiological study on scavenging ducks in smallholder farms in central Java, Indonesia, emphasized that such birds are potentially an important source of H5 virus for native chickens [21]. Most of the previous molecular studies of Indonesian isolates were derived from either chickens or humans. Inadequate data of H5N1 viruses isolated from avian species other than chickens, particularly wild or domestic ducks has meant that little is known about the diversity of H5N1 viruses circulating amongst duck populations in Indonesia. The aim of this study is to characterize H5N1 viruses isolated from 96 smallholder duck farms in central Java, Indonesia between 2007 and 2008. We determined phylogenetic and antigenic relationships of the duck- and chicken-derived H5N1 viruses and we analysed, within the HA and NA genes, the known molecular determinants of pathogenicity, receptor binding, antigenic and catalytic sites, and antiviral susceptibility. We also incorporate field data from a longitudinal survey and disease outbreak investigations in those farms in order to investigate their relationship with the molecular findings.

## Methods

### Sample collection and diagnostic tests

Oropharyngeal and cloacal swabs were collected every two months from individually banded domestic ducks and in-contact chickens during a longitudinal survey conducted between March 2007 and March 2008 on 96 smallholder duck farms in four districts (Magelang, Kulon Progo, Bantul, and Sleman) in central Java, Indonesia [21]. From each bird, the two swabs were placed into a single tube containing 3 ml viral media (Universal Viral Transport, BD-Decton, Dickinson and Company, Franklin Lakes, New Jersey, USA). Samples were also collected during the investigation of bird diseases or bird deaths on the study farms. Oropharyngeal and cloacal swabs were collected from decayed carcasses, while fresh carcasses were transferred to the veterinary diagnostic laboratory at Disease Investigation Centre (DIC) Regional IV Wates, Indonesia, for post-mortem examination and collection of tissue samples. During disease events, the apparently healthy banded birds in the outbreak farms were also swabbed. There was no clinical assessment for birds from which the samples were collected either during the survey or during investigation of diseases; thus, the bird clinical status was only recorded as live or dead.

Molecular and virological testing was conducted in the DIC Wates. Swab media sub-samples from the survey were combined in pools of five by species and tested for the presence of viral RNA using real-time reverse transcription polymerase chain reaction (rRT-PCR) assays for type A influenza and H5 subtype as previously described [22]. Virus isolation in specific-antibody-negative (SAN) embryonated chicken eggs was performed on original rRT-PCR positive or indeterminate swabs collected in the longitudinal survey and on swabs and tissue samples collected during disease investigations. The H5 virus then was confirmed by haemagglutination inhibition (HI) assay with H5-specific antiserum using standard methods [23].

### Virus isolates

Equal numbers of virus isolates from chickens (n = 50) and ducks (n = 50) were selected from 132 samples collected over the study period of 13 months and they were sent to the CSIRO Australian Animal Health Laboratory (AAHL), Geelong, Australia, for molecular and antigenic characterization. These viruses were propagated in specific pathogen-free (SPF) embryonated chicken eggs within microbiological physical containment level 3 facilities at AAHL. Allantoic fluid was collected and tested for haemagglutination of chicken red blood cells (RBC), followed by rRT-PCRs for influenza type A and H5 subtype viruses [22].

Eighty-four samples were found to have viable H5 subtype virus and they were subjected to molecular characterization. Of these 84 viruses, 8 were isolated from dead ducks, 46 from dead chickens, and 28 and 2 were isolated from live ducks and live chickens, respectively. Seventy-six (90.5%) viruses were isolated from live or dead ducks or chickens during the investigation of disease outbreaks, while the remaining eight (9.5%) viruses were isolated from live ducks during the bi-monthly survey. A high proportion of these viruses were collected in July 2007 (19 isolates from 7 farms) and September 2007 (29 isolates from 7 farms), followed by January 2008 (12 isolates from 6 farms) and August 2007 (8 isolates from 3 farms). A lesser number of viruses were isolated from 2-4 farms in May, June, November and December 2007.

### Nucleotide sequencing of the virus isolates

Sequencing of the HA gene was conducted on all 84 virus isolates, while a subset of 24 isolates were selected for NA gene sequencing based on characteristics of their HA amino acid sequence and position in the HA phylogenetic tree. Viral RNA was extracted from allantoic fluids using RNeasy^® ^Mini Kit (Qiagen, Australia) as per manufacturer's protocol. One-step RT-PCR reaction were performed with Super-Script™ III Reverse Transcriptase (Invitrogen, Australia) using respective primers for HA and NA, to obtain overlapping fragments that span the entire coding sequence of each gene. All primers were tagged with M13 compatible sequences to facilitate sequencing (primer sequences available upon request). Conditions for RT-PCR were 48°C for 30 min, followed by 40 cycles of 94°C 30 sec, 54°C 40 sec, and 68°C 40 sec, and final extension 68°C for 5 min. PCR products were extracted from an agarose gel using QIAquick Gel Extraction Kit (Qiagen, Australia), and each purified amplicon was used directly for cycle sequencing using BigDye Terminator^® ^v3.1 Sequencing Kit (Applied Biosystems, Foster City, CA, USA). Post sequencing products were purified using BigDye XT Terminator^® ^Purification Kit (Applied Biosystems, Foster City, CA, USA) prior to running on the ABI PRISM 3130*xl *Genetic Analyzer (Applied Biosystems, Foster City, CA, USA).

The HA and NA nucleotide sequences of the virus isolates reported in this study are available in GenBank database under 108 accession numbers [GenBank: CY091859 to CY091966].

### Phylogenetic and sequence analysis

DNASTAR Lasergene 8.0 sequence analysis software (DNASTAR, Inc., Lasergene, Madison, WI, USA) was used for raw sequence data assembly and editing. Virus gene sequences were aligned using ClustalW program within the Bioedit 7.5 program [24] to compare with representative Indonesian H5N1 influenza A virus sequences that have been published and available on GenBank database [18,19]. Multiple sequence alignments of the HA (1683 bp) and NA (1353 bp) coding sequences, were used for phlylogenetic analysis. To determine the evolutionary relationships of the viruses, phylogenetic analysis was conducted using the Neighbour-Joining (NJ) method provided in the MEGA 4.0 software [25] with 2000 bootstrap replicates and the Tamura-Nei 93 (TN93) for nucleotide substitution model. Clustering within H5N1 clades was investigated by pairwise analysis of HA sequence pairs between and within groups using the same MEGA program. Amino acid sequences were analysed to identify known residues associated with HA receptor binding, antigenic and pathotyping cleavage sites, NA active sites, and sites associated with NA inhibitor susceptibility. H5 numbering [26] used throughout the study was based on the alignment with A/Goose/Guangdong/1/96 (H5N1) minus the 16 amino acids known as HA signal peptide [27]. N1 numbering of the isolates was based on the alignment with the same H5N1 virus, starting from the initiating methionine residue.

### Detection of selection pressure on the HA genes

Potential positive (diversifying) and negative (purifying) selection affecting the HA gene were detected by three codon-based maximum-likelihood methods, single likelihood ancestor counting (SLAC), fixed effects likelihood (FEL), and internal fixed effects likelihood method (IFEL), using the web interface of the HY-PHY package (http://www.datamonkey.org) [28]. A statistical significance of no greater than 0.05 (*p *< 0.05) was used on each method, which meant that less than 5% of neutrally evolving sites may be incorrectly classified as selected [28]. The Akaike's Information Criterion test selected TN93 as the best fitting model of nucleotide substitution in this package; therefore, positive selection (non-synonymous substitution rate higher than synonymous substitutions rate, *d*_N _>*d*_S_) and negative selection (*d*_N _<*d*_S_) were predicted in all test methods using this model.

### Antigenic analysis

The 24 virus isolates that were selected for NA sequencing were further characterized for their antigenic reactivity based on the HI test using a panel of chicken sera produced from two clade 2.1.3 virus antigens, A/chicken/Konawe Selatan/BBVM-204O/2007 (Konawe 204O/07) and A/chicken/Indonesia/Wates1/2005 (Wates1/05) and one clade 1 antigen, A/chicken/Vietnam/08/2004 (Vietnam/08/04). Another serum generated from clade 2.1.3 virus antigen was also used to detect any viruses that were antigenically similar to the recognized antigenic variant, A/chicken/West Java/PWT-WIJ/2006 (PWT-WIJ/06) [29,30].

For the HI assay, 25 μl PBS was added to all wells of a 96-well U-bottom microtiter plate. Each serum was diluted 1:4 in phosphate buffered saline (PBS, pH 7.3), and then added in 25 μl volumes to each well of column 1 and 2 of each test plate. Two-fold serial dilutions of sera were performed from column 3 to 11. Four hemaglutination unit (HAU) in 25 μl of working solution of viral antigen was added into all wells of these columns, but not to column 1 and 12 because they served as serum and RBC controls, respectively. The plates were incubated at 37°C for 30 min. Working solution of antigen was back titrated in a separate plate by 2-fold dilutions. Fifty microlitres of 0.5% chicken RBCs was added to all wells and the plates were incubated at 4°C and read after 45-60 min. The HI titre was determined to be the inverse of the last dilution of sera showing complete inhibition of RBC agglutination. The antigenic pattern of each virus was expressed based on HI titers using the reciprocal value of log2.

## Results and Discussion

### H5 virus infection in smallholder duck farms

In total, 132 H5 subtype viruses were isolated from 46 of the 96 study farms over the 13-month study period (Table 1). H5 virus was first isolated from a live duck, sampled in March 2007 during the bi-monthly survey on a farm in Bantul district. The virus was detected at repeated events (an event is the sampling date from which virus isolations were made, either from the longitudinal survey or from the investigation of diseases) on 17 of these 46 farms. Of these 17 farms, 7 farms (farm no. 1-7) had repeated events detected in single species (only in chickens or ducks), whereas 10 farms (farm no. 8-17) had repeated events detected in both species. The majority of these farms had repeated virus isolations at two different events, with the exception of two farms on which H5 virus could be isolated at three different events (farm no. 9: two events in July 2007 and one event in December 2007, and farm no 14: three events in September and December 2007 and February 2008). On the remaining 29 farms (farm no. 18-46), H5 virus was detected at a single event, mostly in only one species, except for two farms (farm no. 30 and 34) on which H5 viruses were isolated from both chickens and ducks on one day during the outbreak investigation. These results, which showing multiple H5 virus isolations from single farms over different months and in both species, indicate that the virus may circulate over long periods at the flock or farm level. We wished to investigate if these long periods of virus detection resulted from persistence of single variants or introduction of new viral variants (discussed below).

**Table 1:** Details of H5 viruses^a ^isolated from 46 smallholder duck farms in four districts of central Java, Indonesia, March 2007 - March 2008

Farm **no**.	Farm identity	District name	Date of sample collection	Type Visit	Details of event cases
**Repeated events in a single species**					

1	BRIN16	Bantul	27-Jun-07	DI	1 duck (dead)
			04-Feb-08	DI	1 duck (live)
2	BTIR21	Bantul	10-Jul-07	DI	1 chickens (dead)
			18-Jul-07	DI	2 chickens (dead)
3	BTIR22	Bantul	15-Jul-07	DI	1 duck (live)
			30-Jan-08	DI	1 duck (dead)
4	SSDG65	Sleman	31-Jul-07	DI	2 chickens (dead)
			27-Jan-08	DI	2 chickens (dead)
5	SSUM68	Sleman	14-Sep-07	DI	1 chicken (dead)
			08-Mar-08	DI	1 chicken (dead)
6	KBUG31	Kulon Progo	09-Nov-07	DI	1 chicken (dead)
			10-Mar-08	DI	1 chicken (dead)
7	SMAR56	Sleman	26-Jan-08	DI	1 chicken (dead)
			06-Feb-08	DI	3 chickens (dead)

**Repeated events in both species**					

8	BCAT02	Bantul	08-Mar-07	LS	1 duck (live)
			21-Jul-07	DI	1 chicken (dead)
9	BCAT01	Bantul	06-Jul-07	DI	1 chicken (dead)
			12-Jul-07	DI	4 chickens (dead)
			17-Dec-07	DI	1 duck (live)
10	SSDG62	Sleman	21-Jul-07	LS	1 chicken (live)
			24-Jul-07	DI	1 chicken (dead), 1 duck (dead)
11	KBUG35	Kulon Progo	19-Sep-07	DI	4 chickens (dead)
			22-Sep-07	DI	2 chickens (dead), 2 ducks (live)
12	MBUM76	Magelang	19-Sep-07	DI	2 chickens (dead)
			22-Sep-07	DI	1 chickens (dead), 1 duck (live)
13	MBUM75	Magelang	21-Sep-07	DI	2 chickens (dead), 8 ducks (live)
			27-Sep-07	LS	1 chicken (live)
14	SSUM70	Sleman	21-Sep-07	DI	1 duck (dead)
			15-Dec-07	DI	1 chicken (dead)
			18-Feb-08	DI	1 chicken (dead)
15	MDON79	Magelang	27-Nov-07	LS	5 chickens (live)
			14-Feb-08	DI	1 chicken (dead), 2 ducks (live)
16	SMAR60	Sleman	01-Feb-08	DI	2 chickens (dead)
			05-Feb-08	DI	5 ducks (live)
17	SMAR57	Sleman	15-Jan-08	DI	1 chicken (dead), 3 ducks (live)
			22-Jan-08	DI	3 chickens (dead)

**Single event**					

18	BCAT04	Bantul	03-May-07	DI	1 duck (dead)
19	SMAR58	Bantul	15-May-07	LS	2 ducks (live)
20	MBUL95	Magelang	28-May-07	LS	2 ducks (live)
21	BCAT06	Bantul	19-Jun-07	DI	1 duck (dead)
22	BPON12	Bantul	23-Jun-07	DI	3 ducks (live)
23	BRIN17	Bantul	10-Jul-07	DI	1 duck (dead)
24	BRIN18	Bantul	11-Jul-07	DI	1 duck (live)
25	KWAH48	Kulon Progo	13-Jul-07	DI	5 chickens (dead)
26	SBAN52	Sleman	20-Jul-07	LS	2 ducks (live)
27	KJAT42	Kulon Progo	25-Jul-07	DI	1 duck (dead)
28	KWAH46	Kulon Progo	02-Aug-07	DI	1 chicken (dead)
29	BPON09	Bantul	16-Aug-07	DI	2 chickens (dead)
30	KWAH45	Kulon Progo	19-Aug-07	DI	2 chickens (dead), 3 ducks (live)
31	MBUL92	Magelang	25-Aug-07	DI	1 chicken (dead)
32	MBUM74	Magelang	14-Sep-07	DI	4 chickens (dead)
33	SSDG63	Sleman	20-Sep-07	LS	1 chicken (live)
34	BCAT03	Bantul	21-Sep-07	DI	2 chickens (live), 2 ducks (live)
35	MDON81	Magelang	02-Nov-07	DI	1 duck (dead)
36	KJAT41	Kulon Progo	04-Nov-07	DI	2 chickens (dead)
37	BPON10	Bantul	07-Nov-07	LS	1 duck (live)
38	KWAH43	Kulon Progo	26-Nov-07	DI	1 chicken (dead)
39	BCAT05	Bantul	02-Dec-07	DI	1 duck (dead)
40	KBUG36	Kulon Progo	02-Dec-07	DI	1 duck (dead)
41	KJAT39	Kulon Progo	11-Dec-07	DI	1 duck (live)
42	SSDG61	Sleman	16-Dec-07	DI	1 duck (dead)
43	MBUL93	Magelang	15-Jan-08	DI	1 duck (live)
44	KBAN30	Kulon Progo	17-Jan-08	LS	3 ducks (live)
45	SBAN50	Sleman	06-Feb-08	DI	1 chicken (dead)
46	MBUL91	Magelang	26-Mar-08	LS	3 ducks (live)

^a ^The viruses (n = 132) were grouped according to the frequency of isolations (cases) on each farm and to the source species (duck and/or chicken). Two types of visit were conducted on the study farms; bi-monthly longitudinal survey (LS) and investigations of disease outbreaks (DI). An event is the sampling date in which virus isolations were made.

**Figure 1:**
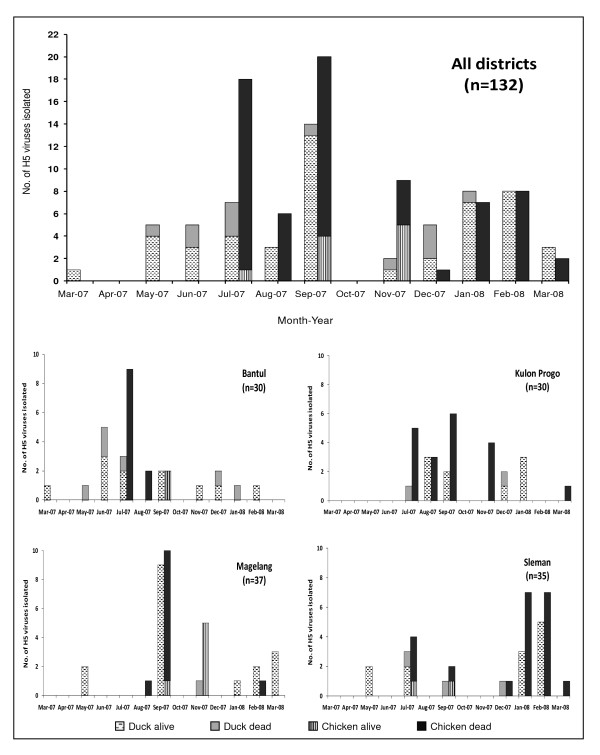
**Temporal distribution of H5 viruses isolated from ducks and chickens in smallholder duck farms in four districts of central Java, Indonesia, March 2007 - March 2008**. H5 virus was isolated in eggs: 1) from individual swab samples of live birds that were monitored in a bi-monthly longitudinal survey and that had H5 positive or indeterminate rRT-PCR pool test results, 2) from individual swab samples of live birds collected during investigations of disease outbreak and 3) from tissue samples of succumbed birds that were collected during investigations of disease outbreaks. The number of virus isolation is shown in parenthesis.

The number of H5 viruses collected from ducks and chickens per month varied over the study period (Figure 1). Throughout the study locations, large numbers of viruses were isolated in some months, for example in July 2007 (7 ducks, 18 chickens) and in September 2007 (14 ducks, 20 chickens), whereas in other months (April and October 2007) no viruses were detected. When examined at the district level, the case numbers constituting these peaks occur mainly in single districts, indicating the relatively localised nature of epidemics. The temporal analysis by species further indicates that while duck cases were more stable over time, chicken cases tended to occur in epidemics, and when present they usually exceeded duck cases in any particular months. It also shows that chicken cases were usually associated with duck cases, but duck cases were often independent.

Although both farm species were affected by HPAI outbreaks, the outcomes of H5 virus infection in ducks seemed to be different to that in chickens. Of the 61 duck-derived viruses, 49 (80.3%) were isolated from live birds, whereas only 10 of 71 (14.1%) chicken-derived viruses were isolated from live birds. Thus, the virus might be more successfully maintained and shed by ducks, often without producing any symptomatic disease. In contrast, a high case fatality rate was always observed in chickens during the HPAI outbreaks, leading also to the more effective reporting of infected chickens compared to ducks. Therefore, where chicken cases occurred they were detected at a higher frequency than in ducks. The increased proportion of live birds, particularly in ducks, being H5 virus positive in certain months coincided with an increase in the number of HPAI outbreaks (July and September 2007) on the same study farms that were reported previously [21]. This is indicative of an increase of live ducks shedding H5 virus during outbreaks. The H5 virus isolation from live chickens suggests that these chickens were in the early stages of HPAI infection, as we could not find these birds in the following survey or disease investigation indicating that they had died or been culled.

### Phylogenetic analysis

**Figure 2:**
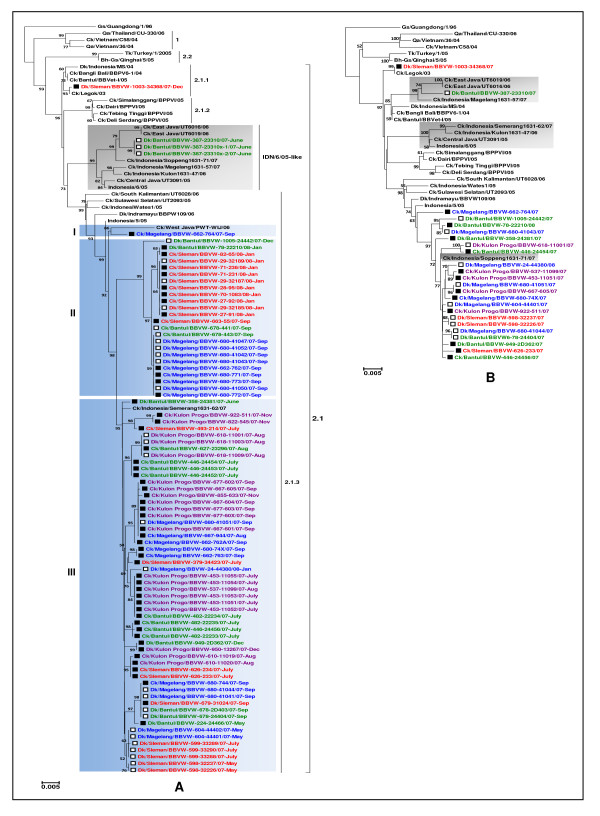
**Phylogenetic relationship of the HA and NA genes of the virus isolates**. The HA (A) and NA (B) phylogenetic trees were constructed by the NJ method with TN93 nucleotide substitution model. Analyses were based on nucleotides 13-1695 and 4-1356 for the HA and NA genes, respectively. Bootstrap values under 50% are not shown. Scale bars indicate number of nucleotide substitutions per site. Viruses isolated from live birds are shown in open squares, while those from dead birds are shown in closed squares. Taxon (virus) names are followed by months when they were isolated and are colour-coded by district of origin: Sleman (red), Bantul (green), Magelang (blue) and Kulon Progo (purple) and sequences obtained from GenBank (black). IDN/6/05-like viruses are shaded in grey. The 2.1.3 clade viruses identified in this study are clustered into three groups; I, II and III (shaded in blue).

The phylogenetic analysis of the HA gene showed that all the H5N1 viruses in the present study belonged to clade 2.1 (Figure 2a). The viruses shared 97-100% nucleotide similarities in the HA gene and 96-100% in the deduced amino acid sequences. The majority of the viruses (80/84) clustered into the third-order clade 2.1.3, one virus belonged to clade 2.1.1, and three viruses were clustered together into a distinct sublineage, which was previously described as Indonesia/6/05 (IDN/6/05)-like viruses [19], whilst no any viruses belonging to clade 2.1.2 were observed in this study. All of the IDN/6/05-like viruses, including three viruses from this study, were descended from a single evident node supported with a high bootstrap statistical value (99%) and had greater than 1.5% average nucleotide distance to other respectively clustered Indonesian H5N1 sublineages recognised as clades 2.1.1, 2.1.2, and 2.1.3. Previous study indicated that the IDN/6/05-like viruses have emerged since 2004 and continue to circulate predominantly in poultry in Java [19].

High phylogenetic relatedness was found amongst the viruses belonging to clade 2.1.3 in this study. Both nucleotide and amino acid sequence identities of these viruses were high (98-100%), indicative of genetic homogeneity. However, it seems the viruses within this clade could further be clustered into three distinct groups, which was supported with high bootstrap value (> 90%), here referred to as group I, II and III (Figure 2a). The majority of these were clustered in group II (24 viruses) and III (55 viruses), while only one virus isolate (Ck/Magelang/BBVW-662-764/07) was situated in group I together with one of the representative viruses that was isolated previously from West Java in 2006. Despite a number of H5N1 sublineages could be identified in this study, we did not observe clear phylogenetic groupings based on the species, clinical status or district origin, which indicates that H5N1 HPAI infection was widespread in the study sites affecting both chickens and ducks.

In relation to district of origin, viruses isolated from Kulon Progo district seemed to have the lowest diversity within district level than the viruses isolated from the three other districts, as they only clustered in group III of clade 2.1.3. Twenty-two viruses isolated from Sleman district were distributed between clade 2.1.1 (1), group II (11), and group III (10). In Bantul district, 3 viruses were classified as IDN/6/05-like viruses, while another 3 and 14 clustered within clade 2.1.3 into groups II and III, respectively. All the viruses originating from Magelang district belonged to clade 2.1.3 with 1, 9 and 11 viruses belonged to the group I, II and III, respectively. Despite the fact that our virus isolates came only from one region in Java and considering the relatively short study period of 13 months, these results suggest that multiple lineages of clade 2.1 viruses have circulated in smallholder backyard farms in central Java and the clade 2.1.3 viruses, in particular, have prevailed amongst poultry on those farms.

H5N1 viruses isolated from ducks appeared to be genetically more diverse than those isolated from chickens. All three virus clades (2.1.1, 2.1.3, IDN/6/05-like) that were identified in this study were isolated from ducks, while only clade 2.1.3 viruses were found in chickens (Figure 2a). We reported previously that H5 RNA was more often detected in live ducks than in live chickens [21], either in the absence or in the presence of antibodies. This implies that H5 virus could circulate more frequently or continuously amongst birds within flocks of ducks. Moreover, duck flocks in our study farms were 12.4 times more likely to have positive antibodies against H5 virus than those in chicken flocks [21] suggesting that ducks are more likely than chickens to harbor antibody to genetically diverse viruses. Previous studies suggested that antigenic variants can be propagated during long-term infection in ducks [9] and antibodies carried by ducks could either protect them against re-infection or exert selection pressure on variants in free-grazing duck populations [31]. Nevertheless, of the 84 viruses analysed in the study, only 4 viruses were from clades other than 2.1.3; therefore, firm conclusions on the species range of the clades cannot be made.

Twenty-four selected viruses had NA nucleotide sequence identity of 96-99%, with the corresponding deduced amino acid sequence similarity of 97-99%. The highest nucleotide divergence (3%) in the NA gene amongst the study viruses was found in Dk/Bantul/BBVW-387-23310/07. For the majority of the viruses that were aligned and analysed, the NA phylogeny corresponded to the HA phylogenetic groupings suggesting concordant evolution of the surface glycoprotein genes amongst the H5N1 viruses examined (Figure 2b). However, we observed placement of some viruses with an IDN/6/05-like HA in different positions within the NA phylogenetic tree. Most of these, including one of our viruses, were clustered into two separate lineages in the NA tree, with the exception of one of the representative clade 2.1 viruses (Ck/Indonesia/Soppeng1631-72/07), which situated outside of those two lineages (Figure 2b, displayed in grey shade). Furthermore, the other representative virus that contained an HA belonged to clade 2.1.3 (Ck/Indonesia/Semerang1631-62/07), clustered into one of those NA lineages with some IDN/6/05-like viruses. These indicate that genetic reassortment events may have occurred on the surface glycoprotein genes of the clade 2.1 viruses.

### Molecular characterization of important sites determining phenotype

High conservation of amino acid sequences was found at the receptor binding site (RBS) of the HA protein. However, some variations were observed at positions 189 (R to K) and 217 (S to P) (Table 2). Three viruses with an IDN/6/05-like HA carried K at position 189 and 218, whereas all the other viruses had 189R and 218K. Binding specificity analysis of A/Vietnam/1203/04 (H5N1) using a glycan microarray showed that K at positions 189 and 218 (193 and 222 in H3 numbering) could increase the interaction of the virus with avian α2-3-linked sialic acid (SA) receptor analogs [32]. Though the mutation of S217P (221 in H3 numbering) in the HA protein of A/Vietnam/1203/04 showed the α2-3 SA receptor binding preference, this mutation could affect specificity of this virus to bind human α2-6 linkage receptors [32]. The S217P substitution was detected in one of our viruses, Ck/KP/607-605/07 (Table 2); thus, further study maybe important to understand the significance of this substitution. Other known mutations linked to increased binding specificity to human-like SA receptors, including Q222L and G224S (226 and 228 in H3 numbering) [32-34], were not observed in all the study viruses, indicating they retained avian receptor-binding characteristics.

**Table 2:** Amino acid variations at critical sites of HA and NA proteins of the H5N1 viruses that determining virus phenotype

		HA^a^																			NA^b^		
	
		Receptor Binding Site		Antigenic site						Glycosylation site			Residues prior to cleavage site								NA-active site	Stalk deletion	Residues resistant to NA inhibitors
																		
Virus name^c^	Status^d^			Site A			Site B	Site E															
								
		189	217	138	140	141	124	83	86	154	155	156	323	324	325	326	327	328	329	330	223	49-68	
Dk/BT/678-24404/07	Live	**R**	**S**	**L**	**S**	**P**	**D**	**A**	**T**	**N**	**S**	**T**	**R**	**E**	**S**	**R**	**R**	**K**	**R**	**R**	**I**	**YES**	**NO**
Dk/BT/678-2D403/07	Live	**-**	**-**	**-**	**-**	**-**	**-**	**-**	**-**	**-**	**-**	**-**	**-**	**-**	**-**	**-**	**-**	**-**	**-**	**-**	nd	nd	nd
Ck/BT/627-23296/07	Dead	**-**	**-**	**-**	**-**	**-**	**-**	**-**	**I**	**-**	**-**	**-**	**-**	**-**	**-**	**-**	**-**	**-**	**K**	**-**	nd	nd	nd
Ck/BT/446-24454/07	Dead	**-**	**-**	**-**	**-**	**-**	**-**	**-**	**I**	**-**	**-**	**-**	**-**	**-**	**-**	**-**	**-**	**-**	**K**	**-**	**-**	**YES**	**NO**
Ck/BT/446-24452/07	Dead	**-**	**-**	**-**	**-**	**-**	**-**	**-**	**I**	**-**	**-**	**-**	**-**	**-**	**-**	**-**	**-**	**-**	**K**	**-**	nd	nd	nd
Ck/BT/446-24453/07	Dead	**-**	**-**	**-**	**-**	**-**	**-**	**-**	**I**	**-**	**-**	**-**	**-**	**-**	**-**	**-**	**-**	**-**	**K**	**-**	nd	nd	nd
Dk/BT/387-23310/07	Live	**K**	**-**	**-**	**T**	**-**	**-**	**V**	**A**	**-**	**-**	**-**	**-**	**-**	**R**	**-**	**-**	**-**	**K**	**-**	**V**	**YES**	**NO**
Dk/BT/387-23310x1/07	Live	**K**	**-**	**-**	**T**	**-**	**-**	**V**	**A**	**-**	**-**	**-**	**-**	**-**	**R**	**-**	**-**	**-**	**K**	**-**	nd	nd	nd
Dk/BT/387-23310x2/07	Live	**K**	**-**	**-**	**T**	**-**	**-**	**V**	**A**	**-**	**-**	**-**	**-**	**-**	**R**	**-**	**-**	**-**	**K**	**-**	nd	nd	nd
Dk/BT/224-24466/07	Dead	**-**	**-**	**-**	**-**	**-**	**-**	**-**	**-**	**-**	**-**	**-**	**-**	**-**	**-**	**-**	**-**	**-**	**-**	**-**	nd	nd	nd
Dk/BT/78-22210/08	Dead	**-**	**-**	**-**	**-**	**-**	**-**	**-**	**-**	**-**	**-**	**-**	**K**	**-**	**-**	**-**	**-**	**-**	**K**	**-**	**-**	**YES**	**NO**
Ck/KP/607-605/07	Dead	**-**	**P**	**-**	**-**	**-**	**-**	**-**	**-**	**-**	**-**	**-**	**-**	**-**	**-**	**-**	**-**	**-**	**K**	**-**	**-**	**YES**	**NO**
Ck/KP/610-11019/07	Dead	**-**	**-**	**-**	**R**	**-**	**-**	**-**	**-**	**-**	**-**	**-**	**-**	**-**	**-**	**-**	**-**	**-**	**K**	**-**	nd	nd	nd
Ck/KP/610-11020/07	Dead	**-**	**-**	**-**	**R**	**-**	**-**	**-**	**-**	**-**	**-**	**-**	**-**	**-**	**-**	**-**	**-**	**-**	**K**	**-**	nd	nd	nd
Dk/KP/618-11001/07	Live	**-**	**-**	**-**	**-**	**-**	**-**	**-**	**I**	**-**	**-**	**-**	**-**	**-**	**-**	**-**	**-**	**-**	**K**	**-**	**-**	**YES**	**NO**
Dk/KP/618-11003/07	Live	**-**	**-**	**-**	**-**	**-**	**-**	**-**	**I**	**-**	**-**	**-**	**-**	**-**	**-**	**-**	**-**	**-**	**K**	**-**	nd	nd	nd
Dk/KP/618-11009/07	Live	**-**	**-**	**-**	**-**	**-**	**-**	**-**	**I**	**-**	**-**	**-**	**-**	**-**	**-**	**-**	**-**	**-**	**K**	**-**	nd	nd	nd
Ck/KP/822-545/07	Dead	**-**	**-**	**-**	**-**	**-**	**-**	**-**	**I**	**-**	**-**	**-**	**-**	**-**	**-**	**-**	**-**	**-**	**K**	**-**	nd	nd	nd
Ck/KP/922-511/07	Dead	**-**	**-**	**-**	**-**	**-**	**G**	**-**	**I**	**-**	**-**	**-**	**-**	**-**	**-**	**-**	**-**	**-**	**K**	**-**	**-**	**YES**	**NO**
Ck/SM/626-233/07	Dead	**-**	**-**	**-**	**R**	**-**	**-**	**-**	**-**	**-**	**-**	**-**	**-**	**-**	**-**	**-**	**-**	**-**	**K**	**-**	**-**	**YES**	**NO**
Ck/SM/626-234/07	Dead	**-**	**-**	**-**	**R**	**-**	**-**	**-**	**-**	**-**	**-**	**-**	**-**	**-**	**-**	**-**	**-**	**-**	**K**	**-**	nd	nd	nd
Dk/SM/679-31024/07	Dead	**-**	**-**	**-**	**-**	**-**	**-**	**-**	**-**	**-**	**-**	**-**	**-**	**-**	**-**	**-**	**-**	**-**	**-**	**-**	nd	nd	nd
Dk/SM/1003-34368/07	Dead	**-**	**-**	**Q**	**K**	**S**	**-**	**-**	**A**	**-**	**-**	**-**	**-**	**-**	**R**	**-**	**-**	**-**	**K**	**-**	**-**	**YES**	**NO**
Ck/SM/82-65/08	Dead	**-**	**-**	**-**	**-**	**-**	**-**	**-**	**-**	**-**	**-**	**-**	**K**	**-**	**-**	**-**	**-**	**-**	**K**	**-**	nd	nd	nd
Ck/MG/662-763/07	Dead	**-**	**-**	**-**	**-**	**-**	**-**	**-**	**-**	**-**	**-**	**-**	**-**	**-**	**-**	**-**	**-**	**R**	**K**	**-**	nd	nd	nd
Ck/MG/680-74X/07	Dead	**-**	**-**	**-**	**-**	**-**	**-**	**-**	**-**	**-**	**-**	**-**	**-**	**-**	**-**	**-**	**-**	**R**	**K**	**-**	**-**	**YES**	**NO**
Dk/MG/680-41041/07	Live	**-**	**-**	**-**	**-**	**-**	**-**	**-**	**-**	**-**	**-**	**-**	**-**	**-**	**-**	**-**	**-**	**-**	**-**	**-**	nd	nd	nd
Dk/MG/680-41044/07	Live	**-**	**-**	**-**	**-**	**-**	**-**	**-**	**-**	**-**	**-**	**-**	**-**	**-**	**-**	**-**	**-**	**-**	**-**	**-**	**-**	**YES**	**NO**
Ck/MG/680-744/07	Dead	**-**	**-**	**-**	**-**	**-**	**-**	**-**	**-**	**-**	**-**	**-**	**-**	**-**	**-**	**-**	**-**	**-**	**-**	**-**	nd	nd	nd
Dk/MG/24-44380/08	Live	**-**	**-**	**-**	**-**	**-**	**-**	**-**	**-**	**-**	**N**	**-**	**-**	**-**	**-**	**-**	**-**	**-**	**K**	**-**	**-**	**YES**	**NO**
Other isolates**^e^**	mix	**-**	**-**	**-**	**-**	**-**	**-**	**-**	**-**	**-**	**-**	**-**	**-**	**-**	**-**	**-**	**-**	**-**	**K**	**-**	**-**	**YES**	**NO**

Amino acid sequence is shown for 30 (HA) and 13 (NA) individual H5N1 viruses. Abbreviation: chicken (Ck), duck (Dk), Bantul (BT), Kulon Progo (KP), Sleman (SM), Magelang (MG), not done (nd).

^a ^H5 numbering is based on the HA protein sequence of A/Goose/Guangdong/1/96 (H5N1) minus the signal peptide.

^b ^N1 numbering starts from the initiating codon residue (methionine) of the NA gene.

^c ^The letters 'BBVW' have been removed from the virus names.

^d ^Status indicates the clinical presentation of birds when samples were collected.

^e ^The remaining isolates had identical sequence in the amino acids of HA (54) and NA (11), respectively.

The maximum-likelihood methods (SLAC, FEL and IFEL) found no positively selected (*p *> 0.05) codon in all amino acid sites in the HA1 and HA2 regions of hemagglutinin of the study viruses. In contrast, several codons appeared to be under negative (purifying) selection (*p *< 0.05) (data not shown). Using the FEL method, 40 codons (24 in HA1 and 16 in HA2) were detected to be restrained by negative selection. The IFEL and SLAC methods were more conservative indicating possible negative selection in only 14 codons (9 in HA1 and 5 in HA2) and 5 codons (2 in HA1 and 3 HA2), respectively. Of the 14 codons predicted to be under possible negative selection using IFEL method, one was located in the putative antigenic site B [31,35] (codon 124, p < 0.046) and the other was in the N-linked glycosylation site [36] (codon 155, p < 0.040).

Despite no evidence of positive selection in the HA of the viruses, amino acid differences were identified at six positions (83, 86, 124, 138, 140 and 141: H5 numbering) within regions homologous to antigenic sites A, B, and E of the H3 HA protein [31,35] (Table 2). Twenty-three viruses (13 chicken and 10 duck) had V to A substitution at amino acid 210, a residue in the putative antigenic site D [31] (data not shown). The four duck viruses outside of clade 2.1.3 (1 belonged to clade 2.1.1 and 3 classified as IDN/6/05-like viruses) possessed more amino acid changes in the other known antigenic sites compared to the clade 2.1.3 majority. All reported IDN/6/05 HA sequences, including our three viruses, possessed a T at position 140, which was not the case for all other HA sequences in this study. In one of our isolates belonging to clade 2.1.1, amino acids Q, K and S were found at position 138, 140 and 141 respectively, which was characteristic of other known clade 2.1.1 viruses. A previous study indicated that five amino acid residues within the HA antigenic sites A and E (positions 83, 86, 138, 140 and 141) of 2002-2005 H5N1 genotype Z influenza viruses from southern China and Southeast Asia were under positive selection pressure [12]. Since we detected no positive selection in the HA sequences of our sample of viruses, the virus population appeared to be stable at this gene. This is expected, as the H5N1 outbreak in Indonesia began about four years prior to the survey. However, it does indicate that there were no significant evolutionary pressures changing the viral population of the backyard and smallholder poultry sectors at this time.

To evade the host's immune defenses, addition of oligosaccharides at certain positions of the HA protein is commonly used by influenza viruses to mask the antigenic epitopes from antibody recognition [36]. Compared to the Eurasian H5N1 progenitor, A/Goose/Guangdong/1/96, an addition of N-linked glycosylation sequence (NST) at the HA protein was detected at position 154 to 156 in all the viruses, except one virus (Dk/MG/24-44380/08) that had S155N substitution (Table 2). Additional glycosylation at this site has been commonly observed in other clade 2.1 viruses. We did not observe extra oligosaccharides at or adjacent to the HA antigenic sites. Furthermore, HA sequence characterization predicted that all the viruses were of the highly pathogenic phenotype, as shown by the presence of multiple basic amino acids at the HA cleavage site sequences [37,38], with 5 motifs observed: RESRRKRR/(7 isolates), RERRRKKR/(4), RESRRRKR/(2), KESRRKKR/(2), and RESRRKKR/(69) (Table 2).

The potential of NA as a target for antiviral therapy has been investigated using NA inhibitors to limit influenza virus infection by blocking the NA enzyme active site [39]. Amino acids relevant to the enzyme-active site of influenza virus neuraminidase [40] were maintained in most of the 24 isolates sequenced (Table 2). One substitution of I223V was found in Dk/BT/387-23310/07 at the NA framework region, which has no direct contact with the substrate sialic acid. NA sequence alignment confirmed a 20-amino acid deletion in the NA stalk region (position 49 to 68: N1 numbering), one of the proposed molecular determinants for the adaptation of influenza viruses from their wild reservoirs to domestic species [41]. Mutations at several recognised NA active sites, including E119V, R152K, D199N, H275Y and R293K, have resulted in resistance of influenza virus to NA inhibitors such as oseltamivir, zanamivir and peramivir [42,43]. Reduced sensitivity to oseltamivir has been previously reported in Indonesian H5N1 viruses isolated from poultry, which sharing I117V and I314V mutations in the NA protein [44]. However, none of the selected viruses in the present study possessed these mutations.

### Antigenic analysis

**Figure 3:**
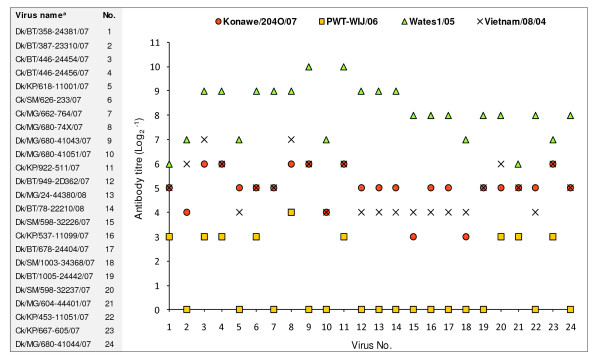
**Antigenic reactivity pattern of a selection of the virus isolates**. Result of HI test of 24 selected isolates against chicken antisera (Konawe/204O/07, PWT-WIJ/06, Wates1/05, and Vietnam/08/04). HI titers are expressed as the reciprocal value of log2. Abbreviation: chicken (Ck), duck (Dk), Bantul (BT), Kulon Progo (KP), Sleman (SM), Magelang (MG). ^a ^The letters 'BBVW' have been removed from the virus names.

Despite natural variations found in the HA antigenic epitopes in some of the viruses, there was no substantial difference in terms of their HI antigenic patterns, indicating that they were antigenically similar (Figure 3). Most of the viruses, including some that had acquired amino acid substitutions at recognised HA antigenic sites, were antigenically closest to A/Ck/Indonesia/Wates1/05, a clade 2.1.3 virus isolated from the same region (central Java) as the study viruses. They had substantial HI reactivity (range of 7 to 9 log2) to Wates1/05 antiserum. Two viruses (Dk/BT/358-24381/07 and Dk/MG/604-44401/07) expressed lower antigenic reactions (6 log2) against this serum, although they were both in clade 2.1.3. In contrast, all the selected viruses demonstrated antigenic difference to antiserum produced against antigenic variant A/Ck/West Java/PWT-WIJ/06 [29,30], with HI titres less than 3 or 4 log2. In response to Konawe/204O/07 antiserum, the viruses showed low to moderate reactivity, ranging from 3 to 6 log2. The clade 1 antiserum, Vietnam/08/04, reacted to some extent (4 to 7 log2) against all selected isolates; this therefore suggests that cross-protection could occur between H5N1 viruses isolated from Vietnam and those isolated from Indonesia.

### Multiple infections of H5N1 virus in smallholder duck farms

On a number of occasions during the 13-month study, multiple H5N1 viruses were isolated from the same farm, either simultaneously or at different sampling points (see above). Amino acid diversity in the HA1 or HA2 molecules of influenza virus hemagglutinin was detected among the viruses from 8 of the 29 farms where multiple viruses were collected (Table 3). On 5 farms (farm no. 4, 9, 13, 32, 34), a substitution of A to V was found in the HA signal peptide in viruses isolated from some, but not all birds on those farms. Most of the isolates had T at position 86, one of the residues in antigenic site E [31,35]. However, T86I substitution was found in 3 of 5 viruses each on farm no. 9 and 30. Another amino acid substitution (S to R) was detected at the HA antigenic site A (position 140) of 2 viruses each on farm no. 30 (of 5 viruses) and farm no. 4 (of 4 viruses). In addition, a S217P change was detected at the RBS of the HA gene in 1 of 6 viruses isolated from farm no. 11 over a 4-day period in September 2007. Amino acid polymorphisms were also observed in the HA cleavage site sequences. Four isolates from farm no. 34 had two different polybasic residues (2 RRKRR/and 2 RRKKR/), 1 of 4 viruses from farm no. 32 had a different sequence motif to the other 3 viruses (RRRKR/and RRKKR/respectively), and 3 motifs (1 RRRKR/, 3 RRKRR/and 6 RRKKR/) were detected among 10 viruses isolated from different birds on farm no.13.

**Table 3:** HA protein sequence diversity of H5N1 viruses associated with HPAI outbreaks in eight smallholder duck farms with multiple isolates

Farm No (Farm ID)	Virus name^a^	Status^b^	Date	HA 1																				HA 2								
					
				-11	-9	-4	66	72	86	95	120	121	140	163	183	210	217	234	236	263	285	328	329	18	62	72	82	143	158	164	183	203
**9**	Ck/BT/446-24454/07	Dead	6-Jul-07	**A**	**L**	**S**	**M**	**N**	**I**	**F**	**S**	**S**	**S**	**S**	**N**	**V**	**S**	**K**	**D**	**A**	**I**	**K**	**K**	**V**	**Q**	**N**	**K**	**K**	**N**	**E**	**T**	**I**
**(BCAT01)**	Ck/BT/446-24452/07	Dead	12-Jul-07	**-**	**-**	**-**	**-**	**-**	**-**	**-**	**-**	**-**	**-**	**-**	**-**	**-**	**-**	**-**	**-**	**-**	**-**	**-**	**-**	**-**	**-**	**-**	**-**	**-**	**-**	**-**	**-**	**-**
	Ck/BT/446-24456/07	Dead	12-Jul-07	**-**	**-**	**-**	**-**	**-**	**T**	**-**	**-**	**-**	**-**	**-**	**D**	**-**	**-**	**-**	**N**	**-**	**-**	**-**	**-**	**-**	**-**	**-**	**-**	**-**	**-**	**-**	**-**	**-**
	Ck/BT/446-24453/07	Dead	12-Jul-07	**-**	**-**	**-**	**-**	**-**	**-**	**-**	**-**	**-**	**-**	**-**	**-**	**-**	**-**	**-**	**-**	**-**	**-**	**-**	**-**	**-**	**-**	**-**	**-**	**-**	**-**	**-**	**-**	**-**
	Dk/BT/1005-24442/07	Live	17-Dec-07	**V**	**-**	**-**	**-**	**-**	**T**	**L**	**-**	**-**	**-**	**G**	**D**	**A**	**-**	**-**	**N**	**T**	**-**	**-**	**-**	**-**	**-**	**-**	**-**	**R**	**-**	**D**	**-**	**-**

**34**	Dk/BT/678-2D403/07	Live	21-Sep-07	**-**	**-**	**-**	**T**	**-**	**T**	**-**	**-**	**-**	**-**	**-**	**D**	**-**	**-**	**-**	**N**	**-**	**-**	**-**	**R**	**-**	**-**	**-**	**-**	**-**	**-**	**-**	**-**	**-**
**(BCAT03)**	Dk/BT/678-24404/07	Live	21-Sep-07	**-**	**-**	**-**	**T**	**-**	**T**	**-**	**-**	**-**	**-**	**-**	**D**	**-**	**-**	**-**	**N**	**-**	**-**	**-**	**R**	**-**	**-**	**-**	**-**	**-**	**-**	**-**	**-**	**-**
	Ck/BT/678-441/07	Live	21-Sep-07	**-**	**-**	**G**	**-**	**-**	**T**	**-**	**N**	**F**	**-**	**-**	**D**	**-**	**-**	**-**	**N**	**-**	**-**	**-**	**-**	**-**	**-**	**-**	**R**	**-**	**S**	**D**	**-**	**-**
	Ck/BT/678-443/07	Live	21-Sep-07	**V**	**I**	**-**	**-**	**-**	**T**	**-**	**-**	**-**	**-**	**-**	**D**	**A**	**-**	**-**	**N**	**-**	**-**	**-**	**-**	**-**	**-**	**-**	**-**	**-**	**S**	**D**	**-**	**-**

**30**	Ck/KP/610-11019/07	Dead	19-Aug-07	**-**	**-**	**-**	**-**	**-**	**T**	**-**	**-**	**-**	**R**	**-**	**D**	**-**	**-**	**-**	**N**	**-**	**-**	**-**	**-**	**-**	**-**	**D**	**-**	**-**	**-**	**-**	**-**	**-**
**(KWAH45)**	Ck/KP/610-11020/07	Dead	19-Aug-07	**-**	**-**	**-**	**-**	**-**	**T**	**-**	**-**	**-**	**R**	**-**	**D**	**-**	**-**	**-**	**N**	**-**	**-**	**-**	**-**	**-**	**-**	**-**	**-**	**-**	**-**	**-**	**-**	**-**
	Dk/KP/618-11001/07	Live	19-Aug-07	**-**	**-**	**-**	**-**	**K**	**-**	**-**	**-**	**-**	**-**	**-**	**-**	**-**	**-**	**-**	**-**	**-**	**-**	**-**	**-**	**-**	**R**	**-**	**N**	**-**	**-**	**-**	**-**	**-**
	Dk/KP/618-11003/07	Live	19-Aug-07	**-**	**-**	**-**	**-**	**K**	**-**	**-**	**-**	**-**	**-**	**-**	**-**	**-**	**-**	**-**	**-**	**-**	**-**	**-**	**-**	**-**	**R**	**-**	**N**	**-**	**-**	**-**	**-**	**-**
	Dk/KP/618-11009/07	Live	19-Aug-07	**-**	**-**	**-**	**-**	**K**	**-**	**-**	**-**	**-**	**-**	**-**	**-**	**-**	**-**	**-**	**-**	**-**	**-**	**-**	**-**	**-**	**R**	**-**	**N**	**-**	**-**	**-**	**-**	**-**

**11**	Ck/KP/667-601/07	Dead	19-Sep-07	**-**	**-**	**G**	**-**	**-**	**T**	**-**	**N**	**F**	**-**	**-**	**D**	**-**	**-**	**-**	**N**	**-**	**-**	**-**	**-**	**-**	**-**	**-**	**R**	**-**	**-**	**-**	**-**	**-**
**(KBUG35)**	Ck/KP/677-602/07	Dead	19-Sep-07	**-**	**-**	**G**	**-**	**-**	**T**	**-**	**N**	**F**	**-**	**-**	**D**	**-**	**-**	**-**	**N**	**-**	**-**	**-**	**-**	**-**	**-**	**-**	**R**	**-**	**-**	**-**	**-**	**-**
	Ck/KP/677-603/07	Dead	19-Sep-07	**-**	**-**	**G**	**-**	**-**	**T**	**-**	**N**	**F**	**-**	**-**	**D**	**-**	**-**	**-**	**N**	**-**	**-**	**-**	**-**	**-**	**-**	**-**	**R**	**-**	**-**	**-**	**-**	**-**
	Ck/KP/677-60X/07	Dead	19-Sep-07	**-**	**-**	**G**	**-**	**-**	**T**	**-**	**N**	**F**	**-**	**-**	**D**	**-**	**-**	**-**	**N**	**-**	**-**	**-**	**-**	**-**	**-**	**-**	**R**	**-**	**-**	**-**	**-**	**-**
	Ck/KP/667-604/07	Dead	22-Sep-07	**-**	**-**	**G**	**-**	**-**	**T**	**-**	**N**	**F**	**-**	**-**	**D**	**-**	**-**	**-**	**N**	**-**	**-**	**-**	**-**	**-**	**-**	**-**	**R**	**-**	**-**	**-**	**-**	**-**
	Ck/KP/667-605/07	Dead	22-Sep-07	**-**	**-**	**G**	**-**	**-**	**T**	**-**	**N**	**F**	**-**	**-**	**D**	**-**	**P**	**-**	**N**	**-**	**-**	**-**	**-**	**-**	**-**	**-**	**R**	**-**	**-**	**-**	**-**	**-**

**32**	Ck/MG/662-762/07	Dead	14-Sep-07	**V**	**I**	**-**	**-**	**-**	**T**	**-**	**-**	**-**	**-**	**-**	**D**	**A**	**-**	**-**	**N**	**-**	**-**	**-**	**-**	**-**	**-**	**-**	**-**	**-**	**S**	**D**	**-**	**-**
**(MBUM74)**	Ck/MG/662-762A/07	Dead	14-Sep-07	**-**	**-**	**G**	**-**	**-**	**T**	**-**	**N**	**F**	**-**	**-**	**D**	**-**	**-**	**-**	**N**	**-**	**-**	**-**	**-**	**-**	**-**	**-**	**-**	**-**	**-**	**-**	**-**	**-**
	Ck/MG/662-763/07	Dead	14-Sep-07	**-**	**-**	**-**	**-**	**-**	**T**	**-**	**-**	**-**	**-**	**-**	**D**	**-**	**-**	**-**	**N**	**-**	**V**	**R**	**-**	**-**	**-**	**-**	**-**	**-**	**-**	**-**	**-**	**-**
	Ck/MG/662-764/07	Dead	14-Sep-07	**V**	**-**	**-**	**-**	**-**	**T**	**-**	**-**	**-**	**-**	**-**	**D**	**-**	**-**	**N**	**N**	**-**	**-**	**-**	**-**	**-**	**-**	**-**	**-**	**-**	**-**	**-**	**-**	**M**

**13**	Ck/MG/680-74X/07	Dead	21-Sep-07	**-**	**-**	**-**	**-**	**-**	**T**	**-**	**-**	**-**	**-**	**-**	**D**	**-**	**-**	**-**	**N**	**-**	**V**	**R**	**-**	**-**	**-**	**-**	**-**	**-**	**-**	**-**	**-**	**-**
**(MBUM75)**	Ck/MG/680-744/07	Dead	21-Sep-07	**-**	**-**	**-**	**T**	**-**	**T**	**-**	**-**	**-**	**-**	**-**	**D**	**-**	**-**	**-**	**N**	**-**	**-**	**-**	**R**	**-**	**-**	**-**	**-**	**-**	**-**	**-**	**-**	**-**
	Dk/MG/680-41042/07	Live	21-Sep-07	**V**	**I**	**-**	**-**	**-**	**T**	**-**	**-**	**-**	**-**	**-**	**D**	**A**	**-**	**-**	**N**	**-**	**-**	**-**	**-**	**-**	**-**	**-**	**-**	**-**	**S**	**D**	**-**	**-**
	Dk/MG/680-41043/07	Live	21-Sep-07	**V**	**I**	**-**	**-**	**-**	**T**	**-**	**-**	**-**	**-**	**-**	**D**	**A**	**-**	**-**	**N**	**-**	**-**	**-**	**-**	**-**	**-**	**-**	**-**	**-**	**S**	**D**	**-**	**-**
	Dk/MG/680-41047/07	Live	21-Sep-07	**V**	**I**	**-**	**-**	**-**	**T**	**-**	**-**	**-**	**-**	**-**	**D**	**A**	**-**	**-**	**N**	**-**	**-**	**-**	**-**	**-**	**-**	**-**	**-**	**-**	**S**	**D**	**-**	**-**
	Dk/MG/680-41050/07	Live	21-Sep-07	**V**	**I**	**-**	**-**	**-**	**T**	**-**	**-**	**-**	**-**	**-**	**D**	**A**	**-**	**-**	**N**	**-**	**-**	**-**	**-**	**-**	**-**	**-**	**-**	**-**	**S**	**D**	**-**	**-**
	Dk/MG/680-41052/07	Live	21-Sep-07	**V**	**I**	**-**	**-**	**-**	**T**	**-**	**-**	**-**	**-**	**-**	**D**	**A**	**-**	**-**	**N**	**-**	**-**	**-**	**-**	**-**	**-**	**-**	**-**	**-**	**S**	**D**	**-**	**-**
	Dk/MG/680-41041/07	Live	21-Sep-07	**-**	**-**	**-**	**T**	**-**	**T**	**-**	**-**	**-**	**-**	**-**	**D**	**-**	**-**	**-**	**N**	**-**	**-**	**-**	**R**	**-**	**-**	**-**	**-**	**-**	**-**	**-**	**-**	**-**
	Dk/MG/680-41044/07	Live	21-Sep-07	**-**	**-**	**-**	**T**	**-**	**T**	**-**	**-**	**-**	**-**	**-**	**D**	**-**	**-**	**-**	**N**	**-**	**-**	**-**	**R**	**-**	**-**	**-**	**-**	**-**	**-**	**-**	**-**	**-**
	Dk/MG/680-41051/07	Live	21-Sep-07	**-**	**-**	**G**	**-**	**-**	**T**	**-**	**N**	**F**	**-**	**-**	**D**	**-**	**-**	**-**	**N**	**-**	**-**	**-**	**-**	**-**	**-**	**-**	**R**	**-**	**-**	**-**	**-**	**-**

**10**	Dk/SM/379-34423/07	Dead	24-Jul-07	**-**	**-**	**-**	**-**	**-**	**T**	**-**	**-**	**-**	**-**	**-**	**D**	**-**	**-**	**-**	**N**	**-**	**V**	**-**	**-**	**-**	**-**	**-**	**-**	**-**	**-**	**-**	**A**	**-**
**(SSDG62)**	Ck/SM/493-214/07	Dead	24-Jul-07	**-**	**-**	**-**	**-**	**-**	**T**	**-**	**-**	**-**	**-**	**-**	**D**	**-**	**-**	**-**	**N**	**-**	**-**	**-**	**-**	**I**	**-**	**-**	**-**	**-**	**-**	**-**	**-**	**-**

**4**	Ck/SM/626-233/07	Dead	31-Jul-07	**-**	**-**	**-**	**-**	**-**	**T**	**-**	**-**	**-**	**R**	**-**	**D**	**-**	**-**	**-**	**N**	**-**	**-**	**-**	**-**	**-**	**-**	**-**	**-**	**-**	**-**	**-**	**-**	**-**
**(SSDG65)**	Ck/SM/626-234/07	Dead	31-Jul-07	**-**	**-**	**-**	**-**	**-**	**T**	**-**	**-**	**-**	**R**	**-**	**D**	**-**	**-**	**-**	**N**	**-**	**-**	**-**	**-**	**-**	**-**	**-**	**-**	**-**	**-**	**-**	**-**	**-**
	Ck/SM/71-231/07	Dead	27-Jan-08	**V**	**I**	**-**	**-**	**-**	**T**	**-**	**-**	**-**	**-**	**-**	**D**	**A**	**-**	**-**	**N**	**-**	**-**	**-**	**-**	**-**	**-**	**-**	**-**	**-**	**S**	**D**	**-**	**-**
	Ck/SM/71-236/07	Dead	27-Jan-08	**V**	**I**	**-**	**-**	**-**	**T**	**-**	**-**	**-**	**-**	**-**	**D**	**A**	**-**	**-**	**N**	**-**	**-**	**-**	**-**	**-**	**-**	**-**	**-**	**-**	**S**	**D**	**-**	**-**

The HA signal peptide that contains 16 amino acids is counted from -15 to 0, using the H5 numbering [26]. Amino acid variations were detected at the HA cleavage site (HA 1, position 328 and 329). Abbreviation: chicken (Ck), duck (Dk), Bantul (BT), Kulon Progo (KP), Sleman (SM), Magelang (MG).

^a ^The letters 'BBVW' have been removed from the virus names.

^b ^Status indicates the clinical presentation of birds when samples were collected.

Based on the HA gene, genetic variations of H5N1 virus were detected in different birds during the same farm outbreak or at different outbreak times (Table 3). On six farms (farm no. 10, 11, 13, 30, 32 and 34) at least two genetic variants were isolated on each farm, either in single or in repeated samplings, during HPAI outbreaks occurring in relatively short time periods, whereas on two farms (farm no. 4 and 9) different variants were detected at 2-3 sampling occasions separated by 5-6 months. Although all of these farms were infected by clade 2.1.3 viruses, phylogenetic analysis showed that some of them (farm no. 4, 9, 13, 32 and 34) were infected by two different virus clusters from groups II and III. These results demonstrate that genetically distinct H5N1 viruses could be isolated from the same farm in multiple, sometimes simultaneous, infections. Conversely, the same genetic variants could be found in HPAI outbreaks on different farms, indicating their wide dispersal.

The presence of multiple genetic variants on a single farm may have resulted from mutations of existing viruses or from introduction of new genetic variants. Mutation was the probable cause of the findings on farm no. 11 (Table 3). Identical HA virus variant was isolated from 4 dead chickens on this farm at the 19 September 2007 outbreak, and a drift mutation was likely to have occurred in this variant, which resulted in the substitution of amino acid (S217P) in one of the viruses isolated from another dead chicken on the same farm three days later, in 22 September 2007 (Table 3). In another case, multiple H5N1 infections seemed to have happened on farm no. 13 during HPAI outbreaks in September 2007. This could be due to an introduction of different virus variants through contact with HPAI infected birds of other flocks during scavenging or through contact with contaminated sources such as traders or farm visitors.

The progress of HPAI spread on some of these farms could be determined by the patterns of HA protein sequence of the related viruses. One of the viruses isolated from a dead chicken on farm no. 32 (Ck/MG/662-762/07) had identical HA sequence to viruses isolated from five live ducks in a later HPAI outbreak on farm no. 13 (both of these outbreaks occurred in September 2007 and were located in the same village in Magelang district) (Table 3). A similar incident of potential transmission of viral variants between these two farms possibly also happened where Ck/MG/662-763/07 (farm no. 32) possessed similar HA sequences to Ck/MG/680-74X/07 (farm no. 13). In farm no 17, identical HA amino acid sequences were found amongst H5N1 viruses isolated from 3 live ducks in 15 January 2008 and those isolated from 3 dead chickens in 22 January 2008 (data not shown). This suggests that surviving ducks could maintain H5N1 virus at least one week in this farm environment, which could lead subsequent HPAI outbreaks. Overall, characterization of HA amino acid sequences showed that the majority of the viruses in these farms possessed T, D and N at residues 86, 183 and 236, respectively (Table 3). Therefore, it is possible that these viruses originated from the same sources, then spread widely in the smallholder duck farms.

We attempted to determine if there was a relationship between clinical outcome of infection and genetic variation. The majority of H5N1 viruses from ducks were isolated from live birds, whereas most chicken infections were lethal, although our sequence analysis included two virus isolates from live chickens and eight from dead ducks. However, multiple protein alignments of the HA gene showed that some viruses isolated from dead ducks or live chickens had identical sequences with other viruses isolated from live ducks or dead chickens (data not shown). The HA phylogenetic analysis also revealed that genetically similar virus could be isolated from birds with different clinical presentations (live or dead). These results indicate that there is no clear correlation between genetic variations or phylogenetic groupings and the pathogenicity of H5N1 virus in these species. This is probably due to the fact that there are other factors that influence pathogenicity.

## Conclusions

In summary, clade 2.1.3 was the dominant circulating H5N1 influenza virus in the smallholder duck farms in central Java between March 2007 and March 2008. Although all the viruses possessed HPAI molecular characteristics with multiple basic amino acids detected at the HA cleavage site, there was no significant amino acid mutations found in either HA or NA proteins, including residues at receptor binding, glycosylation, antigenic and catalytic sites and NA inhibitor targets. Based on the HA gene, identical virus variants could be found at relatively distant and separate geographic locations within the four study districts. Furthermore, genetically distinct variants could be isolated from either chickens or ducks on the same farm at the same time, suggesting that a range of variant viruses can circulate simultaneously within a short period during HPAI outbreaks. Based on the antigenic analysis, there was no evidence of major antigenic variants circulating in these farms during the study period.

The higher proportion of H5 virus isolations from live ducks compared to live chickens suggests that ducks are more resistant to AI virus infection. Some of the viruses in this study were isolated from dead ducks, but there was no clear association of genetic variations with pathogenicity. Whether ducks play a role in the maintenance of Indonesian H5N1 lineage viruses is still unresolved. Therefore, further studies are necessary to investigate other related factors determining pathogenicity in live birds as well as understanding the potential of ducks in maintaining virus infection over long periods.

## Abbreviations

HPAI: Highly pathogenic avian influenza; HA: Hemagglutinin; NA: Neuraminidase; rRT-PCR: Real time reverse transcription polymerase chain reaction; SAN: Specific-antibody negative; SPF: Specific-pathogen free; HI: Haemagglutination inhibition; NJ: Neighbour-Joining; TN93: Tamura-Nei93; SLAC: Single likelihood ancestor counting; FEL: Fixed effects likelihood; IFEL: Internal fixed effects likelihood; RBS: Receptor binding site; SA: Sialic acid; Gs: Goose; Bh-Gs: Bar-headed goose; Dk: Duck; Ck: Chicken; Qa: Quail; Tk: Turkey.

## Competing interests

The authors declare that they have no competing interests.

## Authors' contributions

HW performed the phylogenetic, sequence and antigenic analysis, and had a major role in implementing the HPAI longitudinal survey and outbreak investigation. JH designed the longitudinal study and analyzed the epidemiological results. FW advised on analyzing sequence data and interpreting the phylogenetic findings. PS advised on the analysis and interpretation of the HI data. AJ directed the implementation of the survey. HW, JH, JB, PD and JM implemented and facilitated various aspects of the study. The draft of the manuscript was prepared by HW, with editorial inputs from JH, FW, JB and JM. All authors have read and approved the final manuscript.
